# Feasibility of Xenogeneic Mitochondrial Transplantation in Neuronal Systems: An Exploratory Study

**DOI:** 10.3390/life15070998

**Published:** 2025-06-23

**Authors:** Eriko Nakamura, Tomoaki Aoki, Cyrus E. Kuschner, Yusuke Endo, Jacob S. Kazmi, Tai Yin, Ryosuke Takegawa, Lance B. Becker, Kei Hayashida

**Affiliations:** 1 Laboratory for Critical Care Physiology, Feinstein Institutes for Medical Research, Northwell Health System, Manhasset, NY 11030, USA; 2 Department of Emergency Medicine, Donald and Barbara Zucker School of Medicine at Hofstra/Northwell Health, Hempstead, NY 11549, USA

**Keywords:** mitochondria, mitochondrial transplantation, cardiac arrest, ischemia–reperfusion, neuron

## Abstract

Mitochondrial transplantation (MTx) has emerged as a potential therapeutic approach for diseases associated with mitochondrial dysfunction, yet its scalability and cross-species feasibility remain underexplored. This study aimed to evaluate the dose-dependent uptake and molecular effects of xenogeneic mitochondrial transplantation (xeno-MTx) using rat-derived mitochondria in mouse neuronal systems. HT-22 hippocampal neuronal cells and a murine model of cardiac arrest-induced global cerebral ischemia were used to assess mitochondrial uptake, gene expression, and mitochondrial DNA presence. Donor mitochondria were isolated from rat pectoralis muscle and labeled with MitoTracker dyes. Flow cytometry and confocal microscopy revealed a dose-dependent increase in donor mitochondrial uptake in vitro. Quantitative PCR demonstrated a corresponding increase in rat-specific mitochondrial DNA and upregulation of *Mfn2* and *Bak1*, with no changes in other fusion, fission, or apoptotic genes. Inhibitor studies indicated that mitochondrial internalization may involve actin-dependent macropinocytosis and cholesterol-sensitive endocytic pathways. In vivo, rat mitochondrial DNA was detected in mouse brains post–xeno-MTx, confirming donor mitochondrial delivery to ischemic tissue. These findings support the feasibility of xeno-MTx and its dose-responsive biological effects in neuronal systems while underscoring the need for further research to determine long-term functional outcomes and clinical applicability.

## 1. Introduction

Mitochondria are vital organelles that govern oxidative phosphorylation, metabolic regulation, and programmed cell death, making them indispensable for neuronal survival and function [1]. Dysfunction within mitochondrial networks disrupts cellular homeostasis, compromises neuronal integrity, and is a hallmark of numerous neurological disorders, including neurodegenerative diseases and ischemic injuries. Given the high metabolic demand of neurons, restoring mitochondrial integrity has emerged as a promising therapeutic approach.

Mitochondrial transplantation (MTx), the transfer of viable mitochondria into damaged cells or tissues, is a novel intervention that has shown promise in experimental models for restoring cellular energy metabolism, reducing oxidative stress, and attenuating apoptotic pathways [2,3,4,5,6]. A major challenge for clinical application of MTx is securing scalable mitochondrial sources.

Xenogeneic (cross-species) transplantation (xeno-MTx) offers a promising solution, as advancements such as alpha-gal knockout pigs provide feasible and immunologically compatible options [7,8], making large-scale MTx more practical. While prior studies have demonstrated mitochondrial uptake and partial integration following MTx [9,10,11], few have explored how the dose of transplanted mitochondria influences uptake efficiency or cellular mitochondrial dynamics. This knowledge gap is critical because dosage optimization could improve the efficacy and safety of MTx protocols.

We previously demonstrated that allogeneic MTx with intravenous administration of donor mitochondria improves survival and neurological outcomes in a rat model of asphyxial cardiac arrest, a phenotype of global ischemia and reperfusion [12]. Building on that work, the present study aims to examine the feasibility of xeno-MTx and evaluate how varying doses of donor mitochondria affect their incorporation into recipient neuronal systems. We hypothesized that higher doses of xenogeneic mitochondria would result in increased cellular uptake.

To test this, we isolated mitochondria from rat skeletal muscle and transplanted them into a murine hippocampal neuronal cell line in vitro and into the brains of mice following cardiac arrest in vivo. Although functional integration and metabolic consequences were not assessed in this preliminary study, we provide quantitative data on dose-dependent mitochondrial internalization. These findings offer foundational insight into the dynamics of xeno-MTx and support its future development as a therapeutic strategy for mitochondrial-related neurological diseases.

## 2. Methods

All animal studies were performed using protocols approved by the Institutional Animal Care and Use Committee at our institution and in accordance with National Institutes of Health guidelines.

### 2.1. Donor Mitochondria Isolation and Staining

Donor mitochondria were isolated from the pectoralis major muscle of healthy Sprague-Dawley adult rats (male, 12–16 weeks old, Charles River Production, Wilmington, MA, USA) using a rapid mitochondrial isolation protocol optimized for high yield and functionality, as described previously by McCully et al. [13,14,15]. Previous studies consistently demonstrate the viability and functionality of mitochondria isolated from skeletal muscle using this method [14,15,16,17]. Briefly, 6 tissue samples were collected using a 6 mm biopsy punch and placed in two dissociation tubes, each containing three samples and 7.5 mL of ice-cold homogenization buffer [300 mM sucrose, 10 mM K-HEPES (pH 7.2), 1 mM K-EGTA (pH 8.0), pH = 7.2]. The tissues were homogenized using a gentleMACS dissociator (Miltenyi Biotec Inc., San Diego, CA, USA) and treated with subtilisin A (protease from Bacillus licheniformis, Sigma-Aldrich, St. Louis, MO, USA) for 10 min on ice. The digested homogenate with 0.49% fatty-acid-free BSA homogenate was centrifuged at 750× *g* for 4 min at 4 °C to remove collagen fibers, and the supernatant was filtered sequentially twice through 40 µm mesh filters and subsequently through a 10 µm mesh filter. The filtrate was centrifuged at 9000× *g* for 10 min at 4 °C, and the pellet was resuspended in a staining solution with respiration buffer (RB) [250 mM sucrose, 2 mM KH_2_PO_4_, 10 mM MgCl_2_, 20 mM K-HEPES (pH 7.2), 0.5 mM K-EGTA (pH 8.0)] containing MitoTracker Deep Red (MTDR, 1 mM of final concentration, Thermo Fisher Scientific, Waltham, MA, USA) and incubated for 30 min on ice.

### 2.2. Transmission Electron Microscopy

After mitochondrial isolation, without mitochondrial fluorogenic staining, pellets were washed twice in 0.1 M sodium cacodylate buffer and fixed in a 2% paraformaldehyde, 2% glutaraldehyde, 0.1 M sodium cacodylate fixative solution (Electron Microscopy Sciences, Hatfield, PA, USA). After 48 h, samples were washed three times in 0.1 M sodium cacodylate and postfixed in a 2% osmium tetroxide, 1.5% potassium ferrous cyanide, and 0.1 M sodium cacodylate solution. Samples were then stained *en bloc* with 2% uranyl acetate in 0.1 M sodium cacodylate buffer for 1 h. Samples were dehydrated in sequentially increasing concentrations of ethanol in sodium cacodylate buffer and infiltrated in Epon Resin (EMBed 812 Kit, Electron Microscopy Sciences, Hatfield, PA, USA). Using an ultramicrotome with a diamond knife (EM UC7, Leica Microsystems, Wetzlar, Germany), 80 nm sections were cut and mounted on 300 mesh copper grids (Electron Microscopy Sciences, Hatfield, PA, USA). Imaging was performed on a Hitachi 7500 electron microscope (Hitachi High-Technologies Corporation, Tokyo, Japan) equipped with a NanoSprint12 12-megapixel CMOS digital camera (Advanced Microscopy Techniques, Woburn, MA, USA).

### 2.3. Recipient Neuronal Cell Culture

Mouse hippocampal neuronal cell line HT-22 (SCC129, Sigma-Aldrich, St. Louis, MO, USA) was cultured as a recipient for in vitro MTx. Neurons were seeded at a density of 5 × 10^4^ cells/well onto glass coverslips (NC0343705, Neuvitro, Camas, WA, USA) in 24-well plates (CLS3473, Sigma-Aldrich, St. Louis, MO, USA) containing high-glucose Dulbecco’s Modified Eagle Medium (DMEM) with 10% fetal bovine serum and 1% penicillin–Streptomycin and incubated at 37 °C in 5% CO_2_. For flow cytometry analyses, the neurons were incubated for 24 h to ensure adequate labeling of endogenous mitochondria.

### 2.4. Flow Cytometry Analysis

MTDR-labeled donor mitochondria were co-cultured with recipient neurons. In this study, the recipient neurons were co-cultured with one of three experimental groups: (1) control group (non-MTx): the cells were cultured without exposure to donor mitochondria, serving as the baseline; (2) higher mitochondrial concentration (H-MTx): cells treated with donor mitochondria at a final concentration of 1 mg/mL, resuspended in 500 μL of fresh prewarmed medium. (3) 100× diluted mitochondrial concentration (lower dose, L-MTx): the cells were treated with donor mitochondria at a final concentration of 10 μg/mL, resuspended in 500 μL of fresh prewarmed medium, representing a 100-fold dilution of H-MTx.

For flow cytometry analyses, endogenous mitochondria in neurons were pre-labeled by MitoTracker Green dye (MTG, Thermo Fisher Scientific, Waltham, MA, USA) 24 h prior to MTx in order to distinguish between endogenous and donor mitochondria. A working solution (final concentration: 1 mM) was prepared by dissolving MTG in DMSO. Single-cell suspensions obtained from each group of in vitro xeno-MTx were subjected to flow cytometric analysis. Suspended cells were centrifuged at 300× *g* for 3 min and resuspended in 1 mL of Flow Cytometry Staining Buffer (FCSB, Invitrogen, Carlsbad, CA, USA) containing 1 μL of 4′,6-diamidino-2-phenylindole (DAPI, Vector Laboratories, Burlingame, CA, USA). Following centrifugation at 500× *g* for 5 min, cells were resuspended in 500 μL of FCSB. Data were acquired with BD LSRFortessa (BD Biosciences, San Jose, CA, USA) and subsequently analyzed using FlowJo software 10.8.1 (Becton, Dickinson & Company).

### 2.5. Visualizations for Dose-Dependent Uptake of Donor Mitochondria

To exclusively visualize the integration and localization of exogenously transplanted mitochondria labeled with MTDR, imaging conditions were optimized to avoid signal overlap and ensure unambiguous interpretation of donor mitochondrial uptake. The cells after co-culture were fixed and stained with phalloidin (Thermo Fisher Scientific, Waltham, MA, USA) to label actin filaments, then mounted using a mounting medium containing 4,6-diamidino-2-phenylindole (DAPI). Samples were imaged using an LSM 880 confocal imaging system (Carl Zeiss Meditec AG, Jena, Germany) with Z-stack imaging, providing high-resolution visualization of mitochondrial transfer and enabling the identification of interactions between donor and native mitochondria across multiple focal planes.

### 2.6. Pretreatment with Mitochondrial Internalization Blockers

To investigate the cellular mechanisms involved in xeno-MTx, HT-22 cells were pretreated with pharmacological inhibitors targeting specific endocytic pathways. After 24 h of culture, HT-22 cells were pre-treated for 30 min with one of the following mitochondrial internalization inhibitors: cytochalasin D (an actin polymerization inhibitor, 10 µM), methyl-β-cyclodextrin (MβCD, cholesterol extraction agent, 1 mM), nocodazole (microtubule polymerization inhibitor, 4 ng/mL), or 5-(N-Ethyl-N-isopropyl) amiloride (EIPA, macropinocytosis inhibitor, 10 or 100 µM). All blockers were purchased from Sigma-Aldrich (St. Louis, MO, USA) and dissolved in dimethyl sulfoxide (D2650, Sigma-Aldrich, St. Louis, MO, USA), followed by dilution in culture media at a ratio of ≥1:10,000. The control group was treated with an equivalent molar concentration of DMSO in media for 30 min. Following pre-treatment, the media were removed, and the cells were washed four times with 1 × PBS before adding 500 µL of fresh media to each well. Cells (5 × 10^4^ cells/well) were then co-incubated with MTDR-labeled donor mitochondria. After 24 h of coincubation, the cells were fixed and stained with phalloidin (Thermo Fisher Scientific, Waltham, MA, USA) to label actin filaments, then mounted using a mounting medium containing DAPI. Mitochondrial integration into the cell was quantified as the area of MTDR-positive (number of pixels per cell), calculated using automated counting software (ImageJ 1.46r, National Institutes of Health, Bethesda, MD, USA).

### 2.7. Measurements of Gene Expression

Total RNA was extracted using TRIzol Reagent (Sigma-Aldrich, St. Louis, MO, USA) and reverse transcribed using SuperScript IV VILO™ Master Mix with ezDNase Enzyme (Thermo Fisher Scientific, Waltham, MA, USA). The quantitative real-time PCR was performed using TaqMan Fast Advanced Master Mix (Thermo Fisher Scientific, Waltham, MA, USA) on the LightCycler 480 system (Roche Diagnostics). The primers used are mouse *dynamin-related protein 1* (*Dnm1l*, Mm01342903_m1), *mitochondrial fission 1 protein* (*Fis1*, Mm00481580_m1), *optic atrophy-1* (*Opa1*, Mm01349707_g1), *Mitofusin-1* (*Mfn-1*, Mm00612599_m1), *Mitofusin-2* (*Mfn-2*, Mm00500120_m1), *B-cell lymphoma 2* (*Bcl2*, Mm00477631_m1), *Bcl-2-associated X protein* (*Bax*, Mm00432051_m1), and *Bcl-2 homologous antagonist/killer* (*Bak1*, Mm00432045_m1).

### 2.8. Preparation of DNA and Quantitative Real-Time PCR for mtDNA

In a separate set of experiments, quantitative real-time PCR was employed to differentiate rat-derived mitochondrial DNA from that of mouse-derived neurons. The analysis utilized primers specific to rat *mt-Nd1* and rat *mt-Nd5* genes, alongside mouse-specific counterparts, to reliably distinguish donor mitochondria from endogenous mitochondrial DNA in the recipient neurons.

Recipient neurons were co-cultured with one of three experimental conditions: (1) the control group (non-MTx), in which cells were cultured without exposure to donor mitochondria, serving as a baseline; (2) the higher mitochondrial concentration group (high-MTx), where cells were treated with donor mitochondria at a final concentration of 10 μg/mL; and (3) the lower mitochondrial concentration group, representing a 100-fold dilution of the higher concentration (low-MTx), where cells were treated with donor mitochondria at a final concentration of 0.1 μg/mL, representing a 100-fold dilution of high-MTx.

In this study, different dose ranges of donor mitochondria were employed for specific assays based on the sensitivity and dynamic range of each technique. For confocal microscopy and flow cytometry, a higher concentration was used to ensure a robust fluorescence signal and reliable detection of mitochondrial uptake within individual cells. In contrast, for qPCR analyses, lower concentrations were selected to avoid signal saturation and to enable detection of dose-dependent trends over a wider dynamic range. These doses were optimized empirically to suit the analytical resolution and physiological relevance of each assay and were applied consistently across replicates within each experimental arm.

HT-22 cells were concentrated and purified using the DNAzol™ Reagent (cat no. 10503027, Invitrogen, Carlsbad, CA, USA) and subsequently subjected to real-time PCR according to the manufacturer’s instruction. PCR was performed using TaqMan™ Fast Advanced Master Mix (A44360, Applied Biosystems™, Waltham, MA, USA) on the LightCycler 480 system (Roche Diagnostics, Mannheim, Germany). All primers were commercially available from the company (Thermo Fisher Scientific, Waltham, MA, USA): *Glyceraldehyde-3-phosphate dehydrogenase* (*Gapdh*, TaqMan Assay ID: Mm99999915_g1), mouse *NADH dehydrogenase 1* (*Nd1*, Mm04225274_s1), mouse *NADH dehydrogenase 5* (*Nd5*, Mm04225315_s1), rat *NADH dehydrogenase 1* (*mt-Nd1*, Rn03296764_s1), and rat *NADH dehydrogenase 5* (*mt-Nd5*, Rn03296799_s1).

### 2.9. In Vivo Mouse Model of Cardiac Arrest and Quantitative Real-Time PCR for mtDNA

We performed all instrumentation according to the previously described protocol [18,19]. In brief, adult male 6–14-week-old C57BL/6 mice (weight 20–30 g; Charles River Production, Wilmington, MA, USA) had free access to food and water before experiments. Mice were anesthetized with 4% isoflurane (Isosthesia, Butler–Schein AHS, Dublin, OH, USA), and the trachea was orally intubated with a 20-gauge catheter. Mechanical ventilation (Minivent, Harvard Apparatus, Holliston, MA, USA) was initiated at a rate of 110/min, I:E = 1:1, F_I_O_2_ = 1.0, and TV was maintained at 8–12 mL/kg per minute. Anesthesia was maintained with isoflurane at 2%. During surgical preparation, body temperature was maintained between 37.0 ± 0.5 °C by an incandescent heating lamp and monitored by a transesophageal thermocouple probe. A microcatheter (EZ-1101, BioTime Inc, CA, USA) was inserted into the left femoral vein for drug and fluid administration. The other microcatheter was inserted into the left femoral artery to monitor the blood pressure. Electrocardiograph (ECG) was recorded by needle probe. Cardiac arrest was quickly induced by 100 μL KCl (80 μg/kg) followed by 200 μL room temperature saline flushing, and they were weaned off the ventilator at the same time. Cardiac arrest was confirmed by loss of ECG activity and blood pressure, which usually sharply decreased below 10 mm Hg. Eight minutes after the induction of cardiac arrest, resuscitation via manual chest compression with one finger over the sternum at a rate of 300 to 400 per minute was initiated together with 1 μg/100 μL epinephrine followed by flushing with 200 μL room temperature saline via the left femoral vein, and ventilation was resumed at the rate of 110/min, I:E = 1:1, F_I_O_2_ = 1.0 with TV maintained at 8–12 mL/kg per minute. The return of spontaneous circulation (ROSC) was confirmed by the return of ECG rhythm and an increase in mean arterial pressure > 60 mmHg.

Donor mitochondria (100 μg mitochondrial protein in 50 μL PBS), derived from either rats (xeno-MTx) or mice (allo-MTx), or an equivalent volume of resuspension buffer (RB) as a vehicle control, was intravenously injected into animals upon achieving ROSC. Thigh muscle from healthy adult male 6–14-week-old C57BL/6 mice was used for the in vivo MTx experiments as mouse donor mitochondria. At 5 min after the injection, mice were euthanized for sample collections of the brain.

The harvested brain samples were subjected to DNA purification and real-time PCR according to the manufacturer’s instructions. All primers were purchased from Thermo Fisher: *Glyceraldehyde-3-phosphate dehydrogenase* (*Gapdh*, TaqMan Assay ID: Mm99999915_g1), rat *NADH dehydrogenase 1* (*mt-Nd1*, Rn03296764_s1), and rat *NADH dehydrogenase 5* (*mt-Nd5*, Rn03296799_s1). The expression of *Gapdh* was used for normalizing the expression of both *mt-Nd1* and *mt-Nd5* genes.

### 2.10. Statistical Analysis

Data are presented as the mean ± standard error of the mean (SEM). Prior to parametric analysis, all datasets were assessed for normality using the Shapiro–Wilk test. Statistical analyses were performed using an unpaired two-tailed Student’s *t*-test or one-way analysis of variance (ANOVA) followed by Tukey’s correction for post hoc comparisons. Additionally, a post hoc test for linear trend was applied to evaluate dose-responsiveness. No data points were excluded as outliers. Statistical significance was defined as *p* < 0.05. All analyses were performed using GraphPad Prism (v.10.3.1; GraphPad Software Inc., La Jolla, CA, USA).

## 3. Results

### 3.1. Transmission Electron Microscopy of Isolated Mitochondria

**Figure 1:**
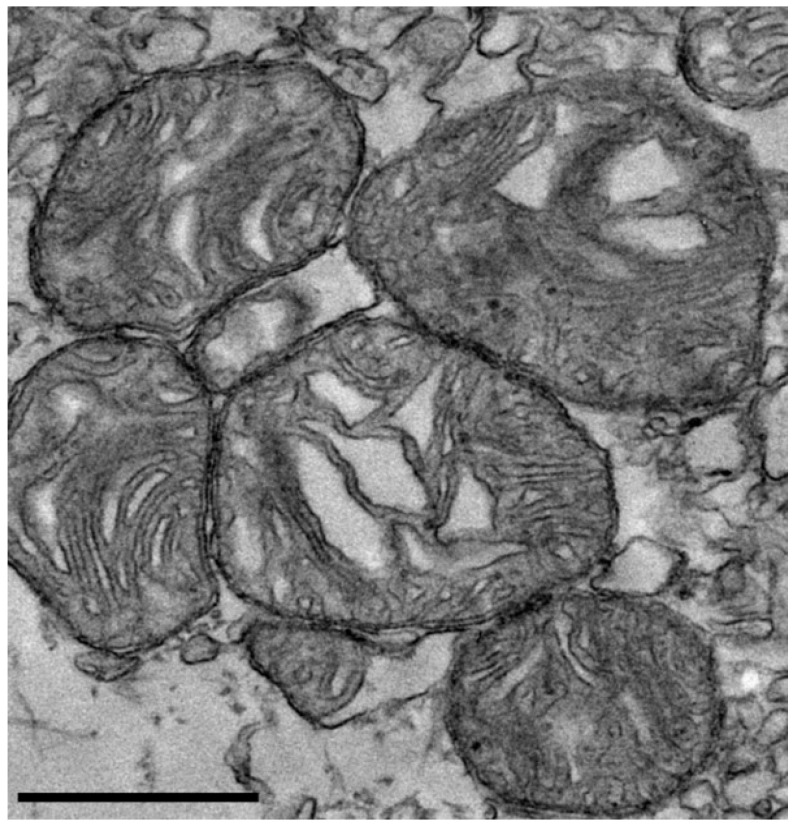
**Transmission electron microscopy of isolated mitochondria**. Representative transmission electron microscopy images showing rat-derived mitochondria with preserved morphology, including intact outer membranes and visible cristae structures. These features are consistent with the structural integrity of the isolated mitochondria. Scale bar = 600 nm.

Transmission electron microscopy of isolated mitochondria revealed preserved morphological features, including intact cristae structure, indicative of structurally viable mitochondria (Figure 1).

### 3.2. In Vitro Uptake of Rat-Derived Donor Mitochondria by Mouse Neurons Across Dosage Groups

**Figure 2:**
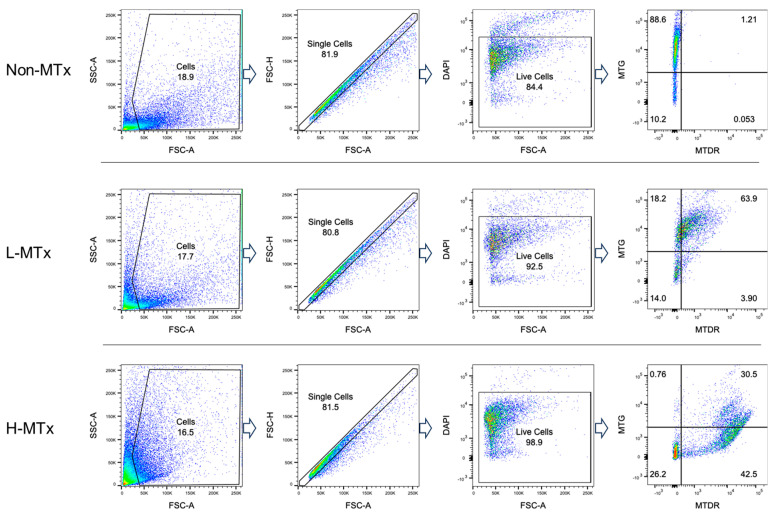
**Representative flow cytometry gating strategy for neuronal cell populations labeled with MitoTracker dyes**. HT-22 murine hippocampal neuronal cells were stained with MitoTracker Green (MTG) to label endogenous mitochondria and MitoTracker Deep Red (MTDR) to label exogenous donor mitochondria. The gating illustrates three distinct cell populations: MTG^+^MTDR^−^ (cells containing only endogenous mitochondria), MTG^+^MTDR^+^ (cells containing both endogenous and donor mitochondria), and MTG^−^MTDR^+^ (cells containing primarily donor mitochondria). Dead cells were excluded by DAPI staining, and fluorescence thresholds were defined using unstained controls.

**Figure 3:**
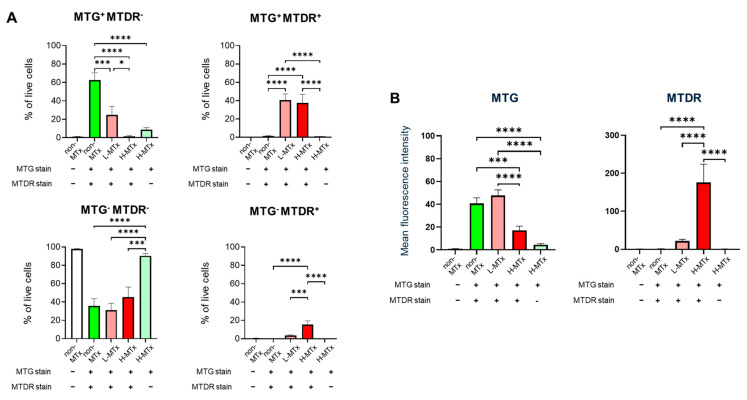
Dose-dependent uptake of donor mitochondria by HT-22 neuronal cells following xenogeneic mitochondrial transplantation. (**A**) Quantitative analysis of HT-22 cell populations shows a dose-dependent decrease in MTG^+^MTDR^−^ cells and corresponding increases in MTG^+^MTDR^+^ and MTG^−^MTDR^+^ cells following exposure to low-dose (L-MTx: 10 µg/mL) and high-dose (H-MTx: 1 mg/mL) donor mitochondria. (**B**) Mean fluorescence intensity (MFI) analysis of MTDR and MTG signals. MTDR MFI increased significantly with higher mitochondrial doses, while MTG MFI decreased following xeno-MTx treatment. Fluorescence intensity was used as a relative surrogate of mitochondrial uptake, although overlap between MTG and MTDR signals may limit interpretability. Data represent mean ± SEM (n = 9 per group). * *p* < 0.05, *** *p* < 0.001, **** *p* < 0.0001 between each group.

Representative flow cytometry plots illustrating the gating process for MTG- and MTDR-positive cell populations were shown in Figure 2. Flow cytometry analysis demonstrated that, among groups with MTG staining, the percentage of MTG^+^MTDR^−^ cells markedly decreased following xeno-MTx and was lower in the L-MTx (10 μg/mL) group than in the H-MTx (1 mg/mL) group. In contrast, the percentage of MTG^+^MTDR^+^ cells significantly increased following xeno-MTx treatment, as did the percentage of MTG⁻MTDR⁺ cells, which was highest in the H-MTx group (n = 9 for each group, Figure 3A). Mean fluorescence intensity (MFI) analysis supported these findings: MTDR MFI was significantly elevated in the L-MTx group compared to the non-MTx control and further increased in the H-MTx group. Conversely, MTG MFI was reduced in MTx-treated groups, consistent with partial displacement or dilution of endogenous mitochondria (n = 9 for each group, Figure 3B). These results suggest a dose-dependent uptake of donor mitochondria by recipient neuronal cells in vitro.

**Figure 4:**
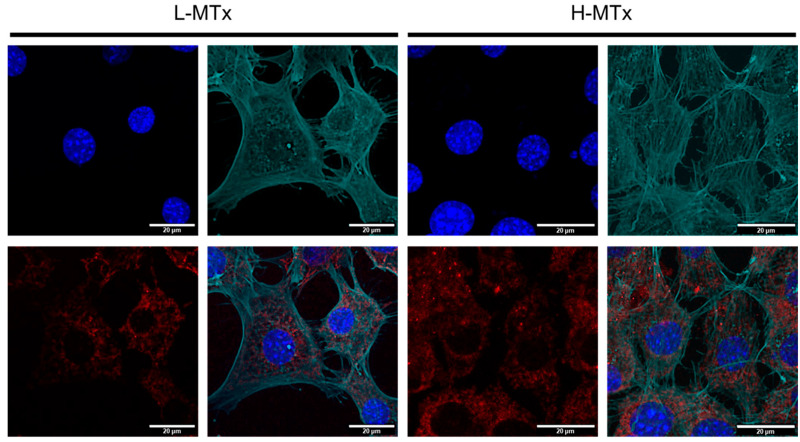
Confocal microscopy showing dose-dependent uptake of donor mitochondria by HT-22 hippocampal neuronal cells. Representative confocal images of HT-22 cells following exposure to low-dose (L-MTx: 10 µg/mL) and high-dose (H-MTx: 1 mg/mL) rat-derived mitochondria labeled with MitoTracker Deep Red (MTDR; red). The actin cytoskeleton is pseudo-colored green to highlight cell morphology and aid in visualizing mitochondrial localization. Nuclei were counterstained with DAPI (blue). Cells treated with L-MTx showed mild-to-moderate MTDR signal, whereas cells treated with H-MTx exhibited more intense and widespread MTDR fluorescence, consistent with a dose-dependent increase in donor mitochondrial presence. Endogenous mitochondria were not labeled in these experiments to avoid signal overlap with exogenous MTDR. Scale bar = 20 μm.

To complement the flow cytometry findings, confocal microscopy was used to visualize MTDR fluorescence within murine hippocampal neurons (Figure 4). The L-MTx group exhibited mild-to-moderate MTDR signal, while the H-MTx group showed more intense and widespread MTDR fluorescence. These observations are consistent with a dose-dependent increase in the presence of donor mitochondria within recipient cells. However, the extent of internalization and the functional integration of these mitochondria were not directly assessed in this study.

### 3.3. Pharmacological Inhibition of Mitochondrial Internalization in HT-22 Cells

**Figure 5:**
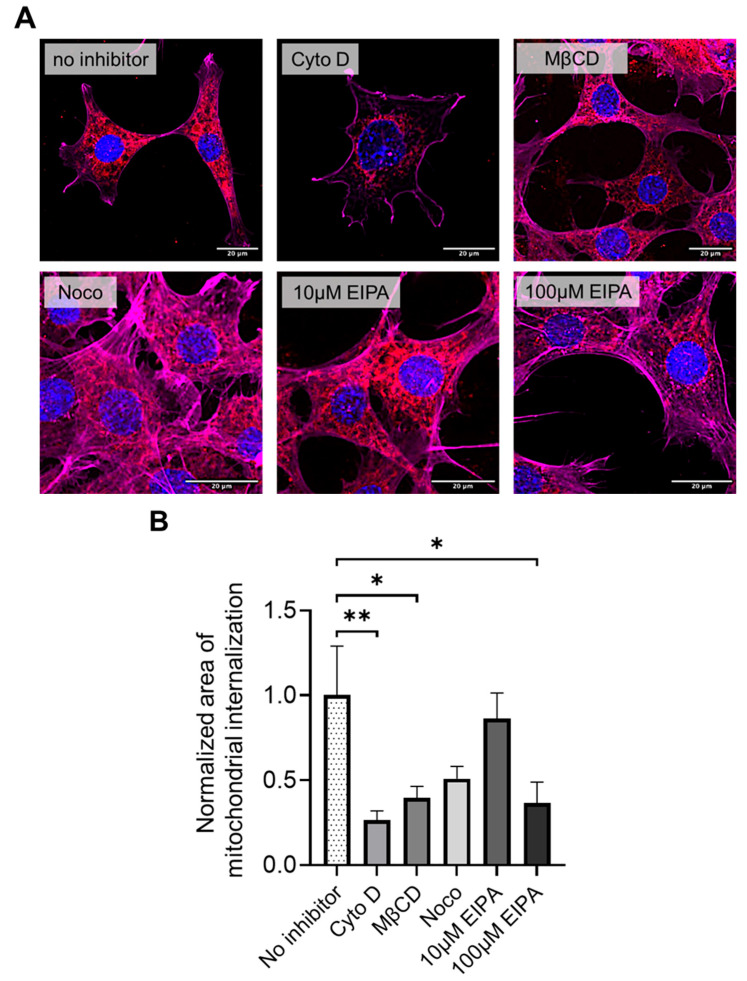
**Pharmacological inhibition of mitochondrial internalization pathways in HT-22 cells during xeno-MTx.** (**A**) Representative fluorescence microscopy images of HT-22 neuronal cells exposed to MTDR-labeled donor mitochondria following pretreatment with pathway-specific inhibitors. Donor mitochondria are visualized in red (MTDR), nuclei in blue (DAPI), and the actin cytoskeleton is pseudo-colored in magenta in this figure (note: pseudo-colored green was used in Figure 4). Reduced MTDR fluorescence was observed after pretreatment with cytochalasin D (actin polymerization inhibitor), methyl-β-cyclodextrin (MβCD; cholesterol-depleting agent), and high-dose EIPA (100 µM; macropinocytosis inhibitor), compared to untreated controls. Pretreatment with nocodazole (microtubule destabilizer) or low-dose EIPA (10 µM) did not markedly affect mitochondrial uptake. (**B**) Quantitative analysis of cellular MTDR signal intensity confirms significant reductions in mitochondrial uptake following treatment with cytochalasin D, MβCD, and high-dose EIPA. Data represent mean ± SEM (n = 12 per group). * *p* < 0.05, ** *p* < 0.01 between each group.

Pharmacological inhibition experiments were performed to explore the mechanisms underlying mitochondrial internalization. HT-22 cells pretreated with cytochalasin D, methyl-β-cyclodextrin (MβCD), and high-dose EIPA (100 µM) exhibited significantly reduced uptake of donor mitochondria compared to untreated controls (n = 12 for each group, Figure 5A,B). In contrast, pretreatment with nocodazole or low-dose EIPA (10 µM) did not significantly affect mitochondrial uptake. These findings suggest that donor mitochondrial internalization during xeno-MTx may involve actin-dependent macropinocytosis and caveolae- or clathrin-mediated endocytosis pathways. Further studies are needed to confirm the specific mechanisms involved.

### 3.4. Real-Time PCR Analysis for In Vitro MTx

**Figure 6:**
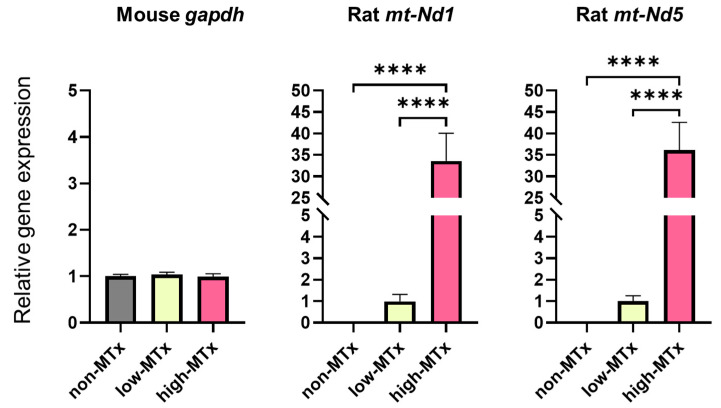
**Detection of rat-specific mitochondrial DNA in recipient neuronal cells following in vitro xeno-MTx.** Quantitative real-time PCR detected rat mitochondrial genes (*mt-Nd1* and *mt-Nd5*) exclusively in HT-22 neuronal cells treated with donor mitochondria (low dose: 0.1 µg/mL; high dose: 10 µg/mL), but not in untreated controls. A statistically significant positive linear relationship was observed between mitochondrial dose and gene copy number for both *mt-Nd1* and *mt-Nd5* (linear trend test, **** *p* < 0.0001). These results are consistent with a dose-dependent increase in the presence of donor mitochondrial DNA in recipient cells. Data are presented as mean ± SEM (n = 6 per group).

Real-time PCR analysis demonstrated that rat mitochondrial genes *Nd1* and *Nd5* were exclusively detected in both low (0.1 μg/mL) and high (10 μg/mL) MTx groups and were absent in the non-MTx control group (n = 6 for each group). Furthermore, a statistically significant positive dose-dependent relationship (linear trend test, *p* < 0.0001) was observed between the relative mRNA expression levels of these genes and the administered mitochondrial dose (Figure 6). These results are consistent with a dose-dependent increase in the presence of donor mitochondrial genetic material within recipient cells. Further studies are warranted to directly evaluate mitochondrial replication, persistence, and functional integration.

### 3.5. PCR for Genes Related with Mitochondrial Dynamics and Apoptosis

**Figure 7:**
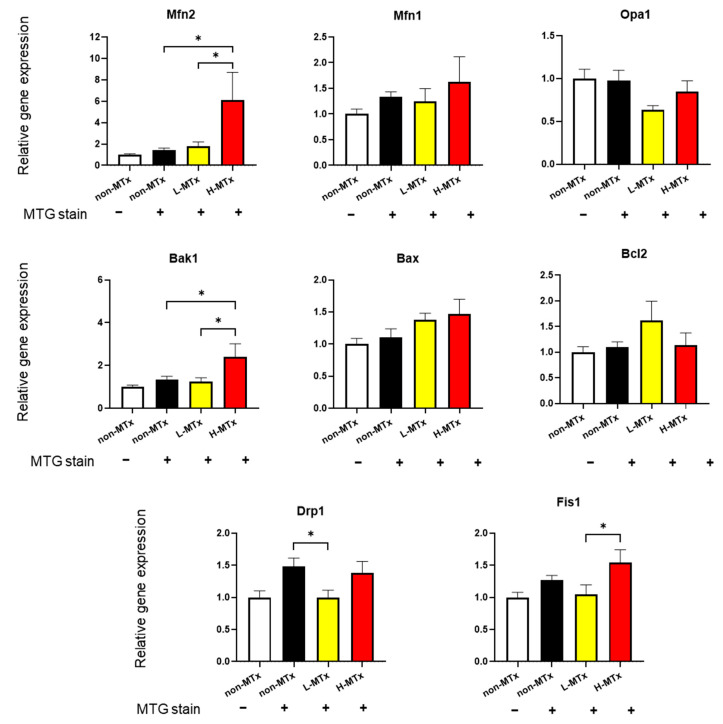
**Effects of xeno-MTx on expression of mitochondrial dynamics and apoptosis-related genes in neuronal cells in vitro**. Quantitative real-time PCR analysis of HT-22 cells treated with low- or high-dose donor mitochondria revealed a dose-dependent increase in the expression of the mitochondrial fusion gene *Mfn2* and the pro-apoptotic gene *Bak1*. No significant dose-dependent changes were observed in other genes related to mitochondrial fission (*Dnm1l*, *Fis1*), fusion (*Opa1*, *Mfn1*), or apoptosis (*Bax*, *Bcl2*). Data represent mean ± SEM (n = 10–13 per group). * *p* < 0.05 between each group.

Gene expression analyses were performed to explore the effects of xeno-MTx on key regulators of mitochondrial dynamics and apoptosis (n = 10–13 for each, Figure 7). While the relative mRNA expression levels of *Dnm1l*, *Fis1*, *Opa1*, and *Mfn1* remained unchanged across groups, *Mfn2* expression showed a statistically significant positive dose-dependent relationship. Similarly, expression of *Bak1*, a pro-apoptotic gene, also increased significantly in a dose-dependent manner. In contrast, there were no significant changes in the expression levels of the anti-apoptotic gene *Bcl2* or the pro-apoptotic gene *Bax*. These findings suggest that xeno-MTx may influence mitochondrial fusion signaling through *Mfn2* and modulate pro-apoptotic gene expression. However, further studies are needed to determine whether these transcriptional changes translate into functional alterations at the protein or cellular level.

### 3.6. Real-Time PCR Analysis for In Vivo Xeno-MTx

**Figure 8:**
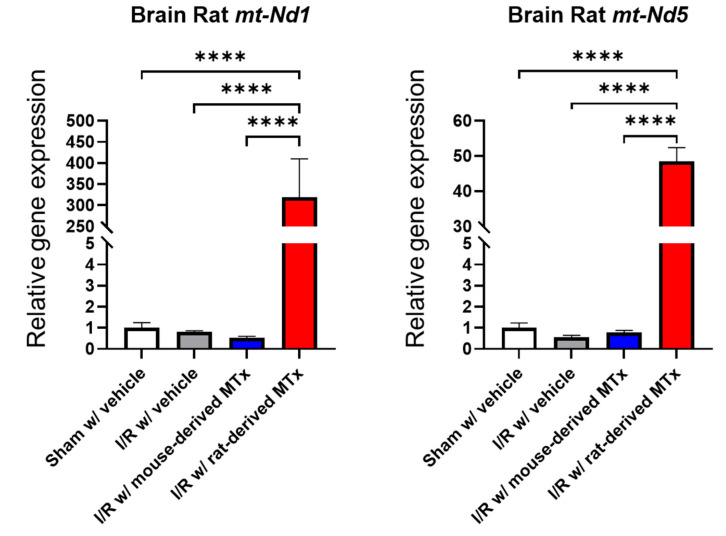
Detection of donor mitochondrial DNA following xeno-MTx in an in vivo mouse model of global cerebral ischemia–reperfusion. Quantitative real-time PCR analysis detected rat-specific mitochondrial genes (*mt-Nd1* and *mt-Nd5*) in the brains of mice that received intravenous donor mitochondria after cardiac arrest and resuscitation. Significantly higher levels of rat mitochondrial DNA were observed in xeno-MTx–treated mice compared to sham-operated controls. These results are consistent with successful delivery and retention of donor mitochondria in ischemic brain tissue. Data presented as mean ± SEM (n = 3–6 per group). **** *p* < 0.0001 between each group.

To assess the feasibility of xeno-MTx in an in vivo model of cardiac arrest and resuscitation, real-time PCR analysis was conducted to detect rat-specific mitochondrial genes (*mt-Nd1* and *mt-Nd5*) in the brains of mice treated with rat-derived donor mitochondria. Donor rat mitochondrial genes were detected at significantly higher levels in MTx-treated mice compared to sham-operated controls (n = 3–6 for each group, Figure 8). These findings are consistent with the presence of donor mitochondrial genetic material in the brain following systemic xeno-MTx. Further investigation is needed to confirm the localization, persistence, and functional relevance of donor mitochondria within recipient brain tissue.

## 4. Discussion

Our findings suggest that xeno-MTx results in dose-dependent uptake of donor mitochondria by recipient neuronal cells. This observation is consistent with previous reports demonstrating the feasibility and potential therapeutic value of mitochondrial transfer across species barriers [20,21,22,23]. The dose-dependent increase in mitochondrial uptake, as quantified by flow cytometry, was further supported by confocal imaging, which showed greater fluorescence intensity in the high-dose group. In vitro real-time PCR analysis detected rat-specific mitochondrial genes in recipient cells, with expression levels positively correlated with mitochondrial dose, supporting the presence of donor-derived mitochondrial genetic material. In vivo, mitochondrial gene detection in brain tissue following cardiac arrest and resuscitation also revealed higher levels of donor mtDNA in MTx-treated mice compared to controls. These findings support the feasibility of systemic xeno-MTx in delivering donor mitochondria to injured brain tissue under ischemic conditions. However, the extent of mitochondrial persistence, localization, and functional contribution remains to be elucidated.

The current study also suggests that mitochondrial uptake during xeno-MTx in HT-22 neuronal cells may involve actin-dependent macropinocytosis and cholesterol-sensitive caveolae- or clathrin-mediated endocytosis pathways. Cytochalasin D, an inhibitor of actin polymerization essential for membrane ruffling and vesicle formation [24,25], significantly reduced donor mitochondrial uptake, implying that actin dynamics contribute to the internalization process. Likewise, pretreatment with MβCD, which disrupts cholesterol-rich lipid raft domains, also inhibited uptake, suggesting a potential role for membrane microdomains in mediating mitochondrial entry. The dose-dependent inhibitory effect of EIPA, particularly at higher concentrations, further supports a role for macropinocytosis in xeno-MTx. As EIPA inhibits Na^+^/H^+^ exchanger-driven macropinocytic activity, these findings suggest that this pathway may be especially important for the internalization of large organelles such as donor mitochondria under xenogeneic conditions [26,27]. In contrast, nocodazole, which disrupts microtubules, did not significantly affect mitochondrial uptake, indicating that microtubule-dependent trafficking may not be involved in the initial stages of xeno-MTx. These preliminary mechanistic insights may reflect differences between xenogeneic and allogeneic MTx. In allo-MTx, where donor and recipient mitochondria are species-matched, prior studies suggest that cytoskeletal elements such as actin filaments and microtubules may facilitate mitochondrial uptake and intracellular trafficking. For example, Pacak et al. [28] demonstrated that mitochondria transplanted into ischemic rat myocardium were rapidly internalized by cardiomyocytes through actin-dependent endocytosis. These findings imply that in allo-MTx, the compatibility between donor and host cell membranes may allow for more efficient internalization via established endocytic pathways. In contrast, xeno-MTx may involve more complex membrane remodeling or alternative uptake mechanisms due to interspecies differences in mitochondrial surface properties. While our study focuses on xeno-MTx, this comparison is intended to provide a conceptual framework and generate hypotheses for future investigation. Understanding such mechanistic differences could help optimize mitochondrial delivery strategies tailored to specific clinical applications.

Gene expression analyses provided supportive evidence for the presence of donor mitochondria within recipient cells. Real-time PCR detected rat-specific mitochondrial genes (*mt-Nd1* and *mt-Nd5*) exclusively in MTx-treated groups, with expression levels increasing in a dose-dependent manner. These results are consistent with the uptake and intracellular retention of donor mitochondrial genetic material following xeno-MTx. In vivo, the detection of rat-derived mitochondrial genes in brain tissue after cardiac arrest and resuscitation further suggests that donor mitochondria can reach and persist within ischemic regions. While these findings indicate successful delivery and molecular persistence of donor mitochondria, additional studies are needed to determine their localization, longevity, and functional impact following transplantation.

In addition to mitochondrial uptake, xeno-MTx appeared to influence the expression of genes related to mitochondrial dynamics and stress signaling. Among the fusion- and fission-related genes assessed, only *Mfn2* demonstrated a significant dose-dependent increase in expression. This observation may suggest that xeno-MTx preferentially promotes mitochondrial fusion while exerting minimal effects on fission-associated pathways. Such a shift toward fusion is consistent with prior studies indicating that enhanced mitochondrial connectivity may support mitochondrial function and cellular resilience under stress conditions [29,30,31]. Interestingly, expression of the pro-apoptotic gene *Bak1* also increased in a dose-dependent manner. Although *Bak1* is classically associated with mitochondrial outer membrane permeabilization and apoptosis, its upregulation may alternatively reflect early apoptotic priming or stress-induced mitochondrial quality control processes. The lack of significant changes in the expression of *Bcl2* and *Bax* suggests that xeno-MTx does not broadly activate apoptosis-related gene networks. Together, these findings may indicate a dose-dependent cellular stress response triggered by higher levels of donor mitochondria, potentially reflecting adaptive remodeling or a threshold for mitochondrial burden. Further studies are needed to determine whether *Bak1* upregulation represents a protective mechanism facilitating selective removal of damaged mitochondria or a maladaptive response associated with early apoptosis.

The detection of rat-derived mitochondrial genetic material in the ischemic mouse brain further supports the feasibility of xeno-MTx as a potential therapeutic approach for neurological disorders [5,23,32,33,34]. The presence of donor mitochondria in brain tissue following cardiac arrest and resuscitation suggests that systemically administered mitochondria can reach injured neural regions and may participate in early cellular responses.

Although the current study did not assess functional outcomes, prior investigations suggest that exogenous mitochondria can improve cellular bioenergetics, attenuate oxidative stress, and promote neuronal survival following ischemic injury [12,35,36,37,38]. These findings collectively highlight the potential of MTx as a platform for metabolic support and underscore the importance of optimizing transplantation parameters—such as dose, timing, and delivery route—for therapeutic application.

To further support this interpretation, our recent study [39] demonstrated that circulating hematopoietic cells can internalize intravenously delivered mitochondria within 30 s after cardiac arrest. This observation provides mechanistic plausibility for the rapid mitochondrial localization observed in the brain at 5 min post-injection in the current study. Nevertheless, qPCR-based detection alone cannot confirm neuronal uptake. To address this, we are conducting follow-up experiments using dual-labeled mitochondria combined with confocal microscopy to visualize and quantify mitochondrial internalization by neurons and glial cells during the early post-resuscitation phase. These ongoing efforts aim to clarify the cell-type specificity and dynamics of xeno-MTx, which will be critical for validating its translational potential.

This study has several limitations. The in vitro neuronal model does not fully reflect the complexity of in vivo systems, where factors such as immune responses, extracellular matrix composition, and organ-specific metabolism may influence mitochondrial uptake, trafficking, and function. Additionally, the dose–response analysis was limited to three groups (control, low, and high dose), which constrains the resolution for determining optimal transplantation parameters. Although mitochondrial gene expression and uptake were evaluated, direct functional assessments—such as measurements of ATP production, oxidative stress, and neuronal viability—were not performed. These analyses will be essential in future studies to comprehensively assess the therapeutic potential and safety profile of xeno-MTx. Finally, despite the promising results, several important challenges must be addressed before xeno-MTx can be translated to clinical applications. Immunological compatibility, donor–recipient mitochondrial mismatch, and species-specific differences in mitochondrial function remain major concerns. Further investigation is needed to evaluate the long-term persistence, functional integration, and immunogenicity of donor mitochondria, as well as their effects on neuronal survival and behavioral outcomes.

## 5. Conclusions

This study suggests that xeno-MTx enables dose-dependent uptake of donor mitochondria by recipient neuronal cells and is associated with modulation of genes involved in mitochondrial fusion and stress response. These findings support the feasibility of xeno-MTx as a delivery strategy and provide a foundation for further investigation. However, since functional outcomes were not evaluated, the therapeutic implications of these molecular changes remain uncertain. Future studies will be essential to determine whether xeno-MTx confers measurable benefits in cellular bioenergetics, neuronal survival, or recovery from ischemic or neurodegenerative injury.

## Data Availability

The datasets used and/or analyzed during the current study are available from the corresponding author on reasonable request.
